# Favorable Outcomes of Anticoagulation With Unfractioned Heparin in Sepsis-Induced Coagulopathy: A Retrospective Analysis of MIMIC-III Database

**DOI:** 10.3389/fmed.2021.773339

**Published:** 2022-01-03

**Authors:** Jiang-Chen Peng, Fang Nie, Yu-Jie Li, Qiao-Yi Xu, Shun-Peng Xing, Wen Li, Yuan Gao

**Affiliations:** Department of Critical Care, Ren Ji Hospital, School of Medicine, Shanghai Jiao Tong University, Shanghai, China

**Keywords:** sepsis-induced coagulopathy, unfractioned heparin (UFH), MIMIC database, survival prognosis, retrospective analysis, propensity score matching

## Abstract

**Backgrounds:** Anticoagulation in sepsis-associated disseminated intravascular coagulation (DIC) remains uncertain. The aim of this study was to investigate whether unfractioned heparin (UFH) could improve clinical outcomes in patients with sepsis-induced coagulopathy (SIC).

**Methods:** Septic patients with SIC were identified from the Medical Information Mart for Intensive Care (MIMIC)-III database. Cox-proportional hazards model, logistic regression model and linear regression were used to assess the associations between UFH administration and 28-day mortality, hospital mortality, occurrence of bleeding complications and length of stay, respectively. Propensity score matching (PSM) analysis was used to match the imbalance between patients in the UFH group and the control group. Patients were further stratified according to SIC score and Simplified Acute Physiology Score II (SAPS II).

**Results:** A total of 1,820 septic patients with SIC were included in the data analysis. After PSM, 652 pairs of patients were matched between the patients in the UFH group and the control group. UFH was significantly associated with reduced 28-day mortality (HR, 0.323, 95% CI, 0.258–0.406; *p* < 0.001) and hospital mortality (HR, 0.380, 95% CI, 0.307–0.472; *p* < 0.001) without increasing the risks of intracranial hemorrhage (OR, 1.480, 95% CI, 0.955–2.294; *p* = 0.080) or gastrointestinal bleeding (OR, 1.094, 95% CI, 0.503–2.382; *p* = 0.820). For subgroup analysis, it didn't change the favorable results of UFH on mortality and UFH didn't increase the risk of hemorrhage in patients with severe disease.

**Conclusions:** The analysis of MIMIC-III database indicated that anticoagulant therapy with UFH may be associated with a survival benefit in patients with SIC.

## Introduction

The activation of coagulation commonly occurs in sepsis as a critical host response to infection that can progress to disseminated intravascular coagulation (DIC) with poor prognosis. In 2001, the International Society of Thrombosis and Haemostasis (ISTH) defined DIC as “an acquired syndrome characterized by the intravascular activation of coagulation with loss of localization arising from different causes that can originate from and cause damage to the microvasculature, which if sufficiently severe, can produce organ dysfunction” (1). Although ISTH overt DIC criteria are widely used, the Japanese Association for Acute Medicine (JAAM) DIC diagnostic criteria are also applied (2). As a result, there doesn't exist gold standard for the diagnosis of DIC currently. What's more, inflammation induced by sepsis is widely considered as a key point in the pathogenesis of coagulation disorder (3), which is characterized by reduced tissue perfusion rather than hypofibrinogenemia (4). However, there were no specific criteria for the diagnosis of sepsis-associated DIC. Therefore, the anticoagulation therapy remains confusing for patients with sepsis-associated DIC based on ISTH or JAAM criteria (5–7).

In 2017, the DIC Scientific and Standardization Committee (SSC) proposed the concept of “sepsis-induced coagulopathy (SIC)” (8). The diagnostic criteria of SIC consists of three items, namely, sepsis-3 definition, platelet count and prothrombin time (PT)–international normalized ratio (INR). SIC was regarded as an earlier phase of DIC due to the reason that SIC included most cases of ISTH overt DIC and SIC developed into overt DIC in every case (9, 10). Thus, the SIC diagnostic criteria may be valuable in identifying septic patients who might benefit form anticoagulant therapy in a timely manner. In this study, we aimed to evaluate the efficacy of unfractioned heparin (UFH) in the treatment of septic coagulopathy based on SIC criteria by retrospective analysis of Medical Information Mart for Intensive Care-III (MIMIC-III) database.

## Materials and Methods

### Data Source

We extracted the data of this retrospective study from MIMIC-III version 1.4 database. MIMIC-III is a large, open, and public database, containing more than 50,000 patients admitted to the CCU at Beth Israel Deaconess Medical Center from 2001 to 2012 (11). Before getting access to the database, “Protecting Human Research Participants” course of the National Institutes of Health has been completed by Jiang-Chen Peng (record ID: 41046393). The establishment and employment of this database were approved by the Institutional Review Boards of the Massachusetts Institute of Technology and Beth Israel Deaconess Medical Center. No informed consent was required since all the data were de-identified.

### Study Population

Patients were eligible if they (1) ≥18 years old; (2) Met the definition of Sepsis 3.0 criteria, which was defined as a suspected infection combined with an acute increase in Sequential Organ Failure Assessment (SOFA) score ≥2 (12). (3) SIC score ≥ 4 (Additional file 1: Supplementary Table 1) according to their worst daily SIC-related values during ICU stay.

The exclusion criteria included, (1) Age < 18 years; (2) ICU stay < 48h; (3) Patients with various cancer types due to their abnormal coagulation function; (4) History of heparin-induced thrombocytopenia; (5) Impaired renal function with estimated glomerular filtration rate (eGFR) <30 ml/min; (6) Concomitant anticoagulant treatment of warfarin; (7) History of embolism and thrombosis; (8) Administration of Low Molecular Weight Heparin (LMWH) due to few cases; (9) Decompensated liver cirrhosis; (10) Missing data >10%.

### Data Extraction

Data were extracted from MIMIC-III database through Structured Query Language (13). For patients with multiple hospitalizations, only the first hospitalization was enrolled. The initial baseline characteristics and laboratory results after admission to ICU were recorded to analyze. The following variables were collected, such as age, gender, laboratory results (white blood cell (WBC) count, platelet count, hemoglobin, INR, partial thromboplastin time (PTT), albumin, total bilirubin (TB), serum creatinine (Scr), pH, partial pressure of oxygen (PO_2_), partial pressure of carbon dioxide (PCO_2_), lactate, anion gap, bicarbonate, serum sodium, serum potassium, serum chloride), infectious sites (lung, abdomen, urine, soft tissue and central nervous system) and mean values of vital signs (mean arterial pressure (MAP), heart rate, temperature and respiratory rate). The comorbidities included hypertension, diabetes mellitus (DM), chronic heart disease (CHD), chronic kidney disease (CKD) and chronic obstructive pulmonary disease (COPD). Treatment contained vasopressor use (epinephrine or norepinephrine), mechanical ventilation and renal replacement therapy (RRT). Clinical severity scales, including SOFA score and Simplified Acute Physiology Score II (SAPS II), were also extracted. For missing variables, predictive mean matching was used to impute numeric features and logistic regression was used for binary variables (Additional file 1: Supplementary Table 2).

### Exposures and Outcomes

Participants were categorized into one of two groups: the UFH group, comprising patients who received UFH subcutaneously or continuous infusion at preventive or therapeutic doses for consecutive at least 5 days (14, 15), and the control group, comprising patients who received no anticoagulant therapy or <5 days.

The primary outcome was 28-day all-cause mortality. Secondary outcomes included hospital mortality, length of ICU stay, length of hospital stay. Bleeding complications such as intracranial hemorrhage and gastrointestinal (GI) bleeding were clarified according to International Classification of Diseases codes version 9 (ICD-9).

### Propensity Score Matching

Propensity score matching (PSM) was used to minimize the effect of confounding factors such as baseline characteristics and disease severity, which may lead to outcome bias. PSM was performed in our study by a nearest neighbor matching using a caliper of 0.05 standard deviations of the logit of the estimated propensity score. Patients were matched in a 1:1 ratio, so that each patient in the UFH group was matched to 1 patient in the control group. The variables shown in Table 1 were all selected to generate the propensity score. Standardized mean difference (SMD) was calculated to evaluate the efficiency of PSM in reducing the differences between the two groups. The primary outcome was further verified by inverse probability of treatment weighted (IPTW), which was created using the estimated propensity scores as weights.

**Table 1:** Baseline characteristics of the study population.

**Variables**	**Control group (***n*** = 1,069)**	**UFH group (***n*** = 751)**	***p*** **-value**	**SMD**
Age, years	67.0 (54.0, 77.0)	64.0 (50.0, 77.0)	0.025	0.135
Male, *n* (%)	613 (57.3%)	449 (59.8%)	0.298	0.050
**Laboratory tests**				
WBC count, (10^3^/μL)	11.9 (8.3, 16.9)	11.9 (8.3, 15.9)	0.319	0.051
Hemoglobin, (g/dL)	10.2 (9.1, 11.4)	10.6 (9.5, 11.8)	<0.001	0.176
Platelet, (10^3^/μL)	159.0 (110.0, 261.0)	172.0 (127.0, 263.0)	0.153	0.068
INR	1.5 (1.4, 1.8)	1.4 (1.3, 1.6)	<0.001	0.186
PTT, (s)	37.5 (30.7, 53.4)	36.1 (29.5, 49.8)	<0.001	0.105
Albumin, (g/dL)	2.6 (2.4, 2.8)	2.6 (2.4, 2.9)	0.054	0.094
Bilirubin, (mg/dL)	1.3 (0.5, 1.9)	0.9 (0.5, 1.9)	0.011	0.107
Scr, (mg/dL)	1.1 (0.8, 1.8)	1.1 (0.8, 1.8)	0.231	0.056
pH	7.37 (7.28, 7.43)	7.35 (7.26, 7.42)	<0.001	0.191
PO_2_, (mmHg)	144.0 (83.0, 240.0)	139.0 (87.0, 223.5)	0.535	0.033
PCO_2_, (mmHg)	42.0 (35.0, 46.0)	42.0 (35.0, 49.0)	0.250	0.045
Lactate, (mmol/L)	2.9 (1.8, 2.9)	2.5 (1.4, 3.0)	0.035	0.116
Anion gap, (mmol/L)	16.0 (13.0, 19.0)	16.0 (14.0, 19.0)	0.512	0.032
Bicarbonate, (mmol/L)	21.0 (18.0, 24.0)	21.0 (17.0, 23.0)	0.108	0.076
Sodium, (mmol/L)	141.0 (138.0, 143.0)	141.0 (138.0, 144.0)	<0.001	0.200
Potassium, (mmol/L)	4.6 (4.1, 5.2)	4.5 (4.2, 5.1)	0.500	0.033
Chloride, (mmol/L)	103.0 (99.0, 107.0)	103.0 (99.0, 108.0)	0.061	0.089
**Vital signs**				
Temperature (°C)	37.0 (36.5, 37.5)	37.1 (36.6, 37.6)	<0.001	0.163
MAP, (mmHg)	75.7 (69.5, 82.9)	76.0 (70.7, 83.5)	0.661	0.019
Heart rate, (min^, −1^)	90.6 (79.9, 102.4)	90.5 (78.1, 102.2)	0.704	0.020
Respiratory rate, (min^, −1^)	19.3 (16.1, 22.9)	19.6 (17.2, 22.9)	0.137	0.072
**Comorbidities**, ***n*** **(%)**				
Hypertension	332 (31.1%)	288 (38.3%)	0.001	0.154
DM	265 (24.8%)	229 (30.5%)	0.007	0.128
CHD	267 (25.0%)	118 (15.7%)	<0.001	0.232
COPD	24 (2.2%)	14 (1.9%)	0.576	0.027
CKD	138 (12.9%)	83 (11.1%)	0.232	0.057
**Source of infection,** ***n*** **(%)**				
Lung	620 (58.0%)	523 (69.6%)	<0.001	0.139
Abdomen	107 (10.0%)	73 (9.7%)	0.839	0.037
Urine	479 (44.8%)	304 (40.5)	0.066	0.085
Soft tissue	41 (3.8%)	47 (6.3%)	0.018	0.102
Central nervous system	8 (0.7%)	9 (1.2%)	0.326	0.062
**Treatment,** ***n*** **(%)**				
Vasopressor	371 (34.7%)	299 (39.8%)	0.026	0.106
Mechanical ventilation	761 (71.2%)	595 (79.2)	<0.001	0.187
RRT	64 (6.0%)	35 (4.7%)	0.219	0.059
**Severity scales**				
SOFA	6 (4, 8)	6 (4, 9)	0.856	0.060
SAPSII	43 (34, 54)	42 (33, 52)	0.187	0.059

*Values were shown as median (interquartile range) unless otherwise indicated*.

*WBC, white blood cell; INR, international normalized ratio; PTT, partial thromboplastin time; Scr, serum creatitine; PO_2_, partial pressure of oxygen; PCO_2_, partial pressure of carbon dioxide; MAP, mean arterial pressure; DM, diabetes mellitus; CHD, coronary heart disease; COPD, chronic obstructive pulmonary disease; CKD, chronic renal disease; RRT, renal replacement therapy; SMD, standardized mean difference; SOFA, Sequential Organ Failure Assessment; SAPSII, Simplified Acute Physiology Score II*.

### Statistical Analysis

Continuous variables were presented as mean ± standard deviation (SD) for normal distribution or medians and interquartile range (IQR) for skewed distribution. Comparisons were made by unpaired Student's test or Mann–Whitney *U*-test, respectively. Categorical data were expressed as frequency (percentage) and were compared by chi-square test or Fisher's exact test, as appropriate. The 28-day survival curves were generated using the Kaplan–Meier method and compared by the log-rank test. The overall associations between UFH treatment and mortality outcomes were evaluated using a Cox-proportional hazards model with hazard ratio (HR) and 95% confidence interval (CI) after adjusting for confounders based on *p*-values < 0.05 in univariate analysis and clinical experience. Impact of UFH use on the risk of hemorrhage events was estimated by bivariate logistic regression model with odds ratio (OR) and 95% CI, which was adjusted for age, gender, platelet count, INR, PTT, SOFA score and SAPS II. Linear regression was used to examine the association between UFH administration and length of stay with β coefficient and 95% CI, which was adjusted for age, gender, SOFA score and SAPS II. PSM and IPTW was used to adjust covariates to ensure the robustness of our findings. To reduce the impact of survivor bias, additional subgroup analyses by stratification to SIC score (4, 5 and 6) and SAPS II (<40 and ≥40) were also performed to explore whether the impact of UFH use on clinical outcomes differed across these subsets. All statistical data were analyzed by SPSS software (v22.0; IBM, Armonk, NY) and R 3.5.3 software for windows. A two-tailed *p* < 0.05 was considered statistically significant.

## Results

### Baseline Characteristics

**Figure 1:**
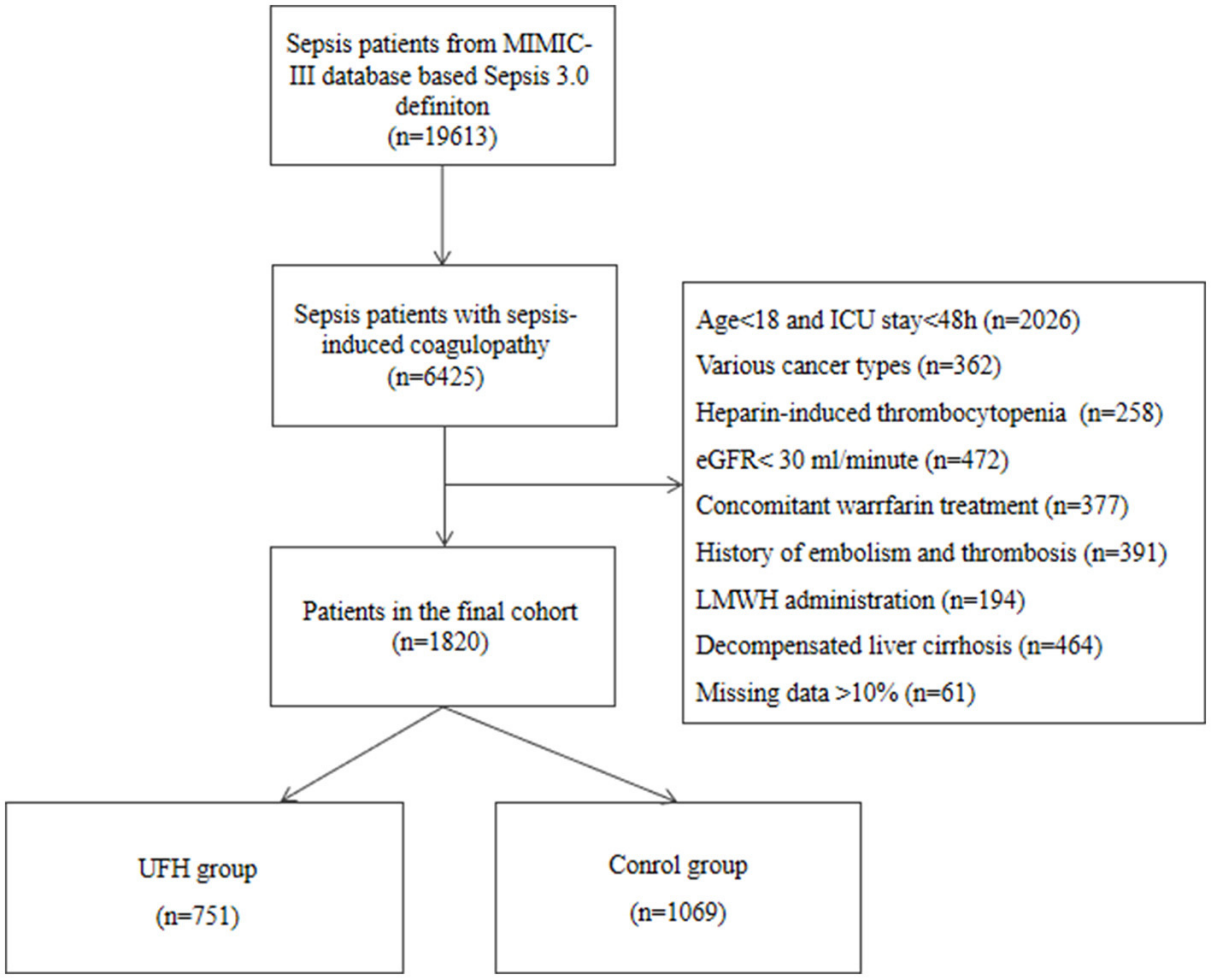
Flow chart of the population included in the study.

A total of 19,613 septic patients were reviewed, of whom 6,425 (32.7%) developed SIC. According to the exclusion criteria, 1,820 eligible patients were included in the final cohort. Then, 751 patients were allocated to the UFH group and 1,069 patients were allocated to the control group. The flow diagram of patient selection is presented in Figure 1. As shown in Table 1, median age seemed to be younger in the UFH group compared with the control group (64.0, 50.0–77.0 vs. 67.0, 54.0–77.0, *p* < 0.025). Gender, SOFA score and SAPS II didn't show significant differences between the two groups. As regarded to laboratory results, most of the laboratory indicators were similar between the two groups, except hemoglobin, INR, PTT, pH and sodium. The proportions of hypertension and DM were greater in the UFH group. In terms of source of infection, the UFH group had higher prevalence of pulmonary infection compared with the control group (69.6% vs. 58.0%, *p* < 0.001). Vasopressor use (39.8%) and mechanical ventilation (79.2%) were more common among patients in the UFH group.

### Clinical Outcomes

**Figure 2:**
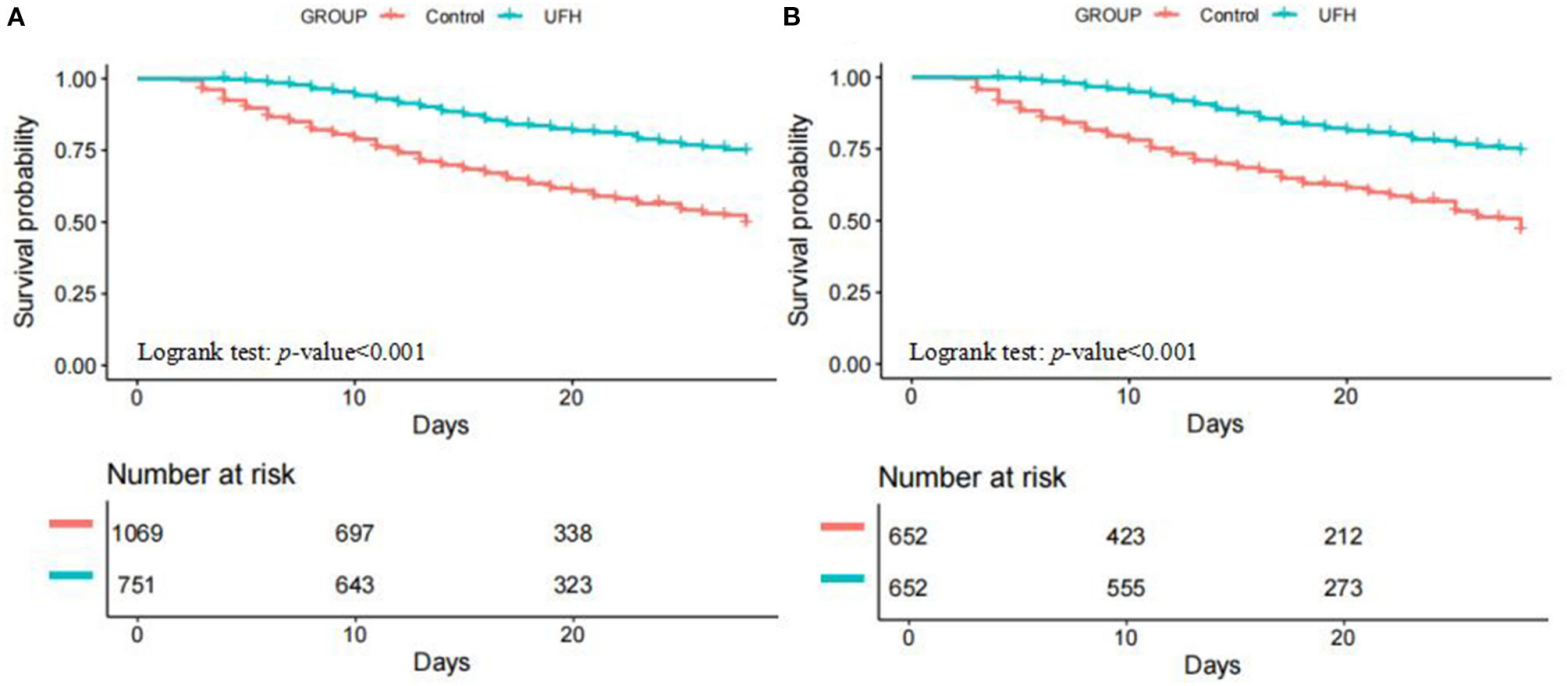
Kaplan–Meier estimates of cumulative probabilities of 28-day survival for SIC patients in UFH group and control group. **(A)** pre-matched cohort; **(B)** post-matched cohort. *SIC, sepsis-induced coagulopathy*.

Table 2 depicted the clinical outcomes of the two groups. As regard to the primary outcome, the UFH group had significantly lower 28-day mortality compared with the control group (17.0% vs. 36.4%, *p* < 0.001). The 28-day Kaplan-Meier survival curves also showed that patients in the UFH group had higher survival probability compared to the control group (*p* < 0.001) (Figure 2A). For hospital mortality, it was still significantly lower in the UFH group compared with the control group (19.7% vs. 38.1%, *p* < 0.001). In terms of bleeding complications, patients in the UFH group had higher occurrence of intracranial hemorrhage (9.1% vs. 4.7%, *p* = 0.002) in comparison with the control group. While, the UFH group had longer length of stay in ICU (11.3, 95% CI, 6.9–18.1 vs. 6.8, 95% CI, 4.2–12.1; *p* < 0.001) and hospital (17.4, 95% CI, 12.2–27.6 vs. 13.2, 95% CI, 8.5–22.3; *p* < 0.001) than that in the control group.

**Table 2:** Association between UFH use and clinical outcomes in patients with sepsis- induced coagulopathy.

**Pre-matched cohort**	**Control grou***p*** (***n*** = 1,069)**	**UFH group (***n*** = 751)**	***p*** **-value**	**Effect size (95% CI)**	***p*** **-value**
28-day mortality^a^	389 (36.4%)	128 (17.1%)	<0.001	HR = 0.361 (0.294, 0.442)	<0.001
Hospital mortality^a^	407 (38.1%)	148 (19.7%)	<0.001	HR = 0.414 (0.314, 0.502)	<0.001
Length of ICU stay, days^b^	6.8 (4.2, 12.1)	11.3 (6.9, 18.1)	<0.001	β = 4.472 (3.539, 5.406)	<0.001
Length of hospital stay, days^b^	13.2 (8.5, 22.3)	17.4 (12.2, 27.6)	<0.001	β = 3.409 (1.966, 4.852)	<0.001
Intracranial hemorrhage^c^	50 (4.7%)	68 (9.1%)	0.002	OR = 1.933 (1.317, 2.837)	<0.001
Gastrointestinal bleeding^c^	19 (1.8%)	17 (2.3%)	0.463	OR = 1.320 (0.673, 2.589)	0.419
**Post-matched cohort**	Control group (*n* = 652)	UFH group (*n* = 652)			
28-day mortality	246 (37.7%)	110 (16.9%)	<0.001	HR = 0.323 (0.258, 0.406)	<0.001
Hospital mortality	251 (38.5%)	128 (19.6%)	<0.001	HR = 0.380 (0.307, 0.472)	<0.001
Length of ICU stay, days	7.0 (4.5, 13.1)	11.1 (6.8,17.8)	<0.001	β = 3.660 (2.495, 4.767)	<0.001
Length of hospital stay, days	13.2 (7.4, 23.1)	17.5 (12.6, 26.2)	<0.001	β = 3.479 (1.849, 5.162)	<0.001
Intracranial hemorrhage	37 (5.7%)	54 (8.3%)	0.065	OR = 1.480 (0.955, 2.294)	0.080
Gastrointestinal bleeding	13 (2.0%)	14 (2.1%)	0.814	OR = 1.094 (0.503, 2.382)	0.820

*Values are shown as median (interquartile range) or n (%) unless otherwise indicated*.

a *Cox regression was used for estimating the impact of UFH use on mortality outcomes after adjusting for confounding variables selected based on p-value < 0.05 in univariate analysis and clinical experience. Results were given as hazard ratio (HR) and 95% confidence interval (CI)*.

b *Linear regression model was used to evaluate the impact of UFH use on length of stay after adjusting for age, gender, SOFA score and SAPSII. Results were given as beta coefficient and 95% CI*.

c *Bivariate logistic regression was used to assess the associations between UFH use and bleeding complications after adjusting for age, gender, platelet count, INR, PTT, SOFA score and SAPSII. Results were given as odds ratio (OR) and 95% CI*.

### Relationship Between UFH and Clinical Outcomes

The univariate analysis of Cox-proportional hazards models between baseline variables and 28-day mortality were listed in Additional file 1: Supplementary Table 3. After adjusting for confounders with *p*-values < 0.05 in univariate analysis, UFH therapy was significantly associated with reduced 28-day mortality (adjusted HR, 0.361, 95% CI, 0.294–0.442; *p* < 0.001) and hospital mortality (adjusted HR, 0.414, 95% CI, 0.314–0.502; *p* < 0.001). However, the logistic regression models found that the administration of UFH was associated with increased incidence of intracranial hemorrhage (adjusted OR, 1.933, 95% CI, 1.317–2.837; *p* < 0.001) but not with GI bleeding (adjusted OR, 1.320, 95% CI, 0.673–2.589; *p* = 0.419). While, UFH use was associated with longer length of ICU stay (adjusted β, 4.472, 95% CI, 3.539–5.406; *p* < 0.001) and hospital stay (adjusted β, 3.409, 95% CI, 1.966–4.852; *p* < 0.001) (Table 2).

### Outcomes After Propensity Score Matching

In PSM, 652 patients in the UFH group were matched with 652 patients in the control group. After matching, the baseline profiles were well balanced between the two groups with SMDs <5% for all variables (Table 3, Additional file 1: Supplementary Figure 1). Similar to the results in the pre-matched cohort, 28-day Kaplan-Meier survival curves also showed that at any instance during the first 28 days after ICU admission, patients in the UFH group were less likely to die than those in the control group (*p* < 0.001) (Figure 2B). Both PSM (HR, 0.323, 95% CI, 0.258–0.406; *p* < 0.001) and IPTW (HR, 0.354, 95% CI, 0.286–0.439) indicated that UFH use was significantly associated with reduced 28-day mortality. As regard to bleeding complications, UFH use was not associated with the occurrence of either intracranial hemorrhage (OR, 1.480, 95% CI, 0.955–2.294; *p* = 0.080) or GI bleeding (OR, 1.094, 95% CI, 0.503–2.382; *p* = 0.820) after PSM. Still, UFH use was associated with longer length of stay in ICU and hospital (Table 2).

**Table 3:** Comparisons of the covariates after propensity score matching.

**Variables**	**Control group** **(***n*** = 652)**	**UFH group** **(***n*** = 652)**	**SMD**
Age, years	65.0 (51.0, 76.0)	65.0 (51.0, 77.2)	0.017
Male	381 (58.4%)	385 (59.0%)	0.016
**Laboratory tests**			
WBC count, (10^3^/μL)	11.9 (8.4, 16.9)	11.9 (8.3, 16.0)	0.033
Hemoglobin, (g/dL)	10.5 (9.4, 11.8)	10.6 (9.4, 11.7)	0.021
Platelet, (10^3^/μL)	159.5 (112.0, 259.0)	172.0 (127.0, 262.0)	0.012
INR	1.4 (1.4, 1.7)	1.4 (1.4, 1.6)	0.004
PTT, (s)	35.7 (29.8, 49.6)	36.4 (29.8, 50.9)	0.004
Albumin, (g/dL)	2.6 (2.4, 2.9)	2.6 (2.4, 2.9)	0.029
Bilirubin, (mg/dL)	1.1 (0.5, 1.9)	0.9 (0.5, 1.9)	0.010
Scr, (mg/dL)	1.1 (0.8, 1.8)	1.1 (0.8, 1.8)	0.029
pH	7.37 (7.29, 7.43)	7.35 (7.26, 7.42)	0.007
PO_2_, (mmHg)	138.0 (81.0, 238.2)	142.5 (86.0, 221.5)	0.023
PCO_2_, (mmHg)	42.0 (35.0, 46.0)	42.0 (35.0, 48.0)	0.012
Lactate, (mmol/L)	2.9 (1.7, 2.9)	2.5 (1.4, 3.0)	0.006
Anion gap, (mmol/L)	16.0 (13.0, 19.0)	16.0 (14.0, 19.0)	0.024
Bicarbonate, (mmol/L)	21.0 (17.0, 24.0)	21.0 (17.0, 24.0)	0.004
Sodium, (mmol/L)	141.0 (138.0, 144.0)	141.0 (138.0, 144.0)	0.013
Potassium, (mmol/L)	4.5 (4.1, 5.1)	4.5 (4.2, 5.1)	0.022
Chloride, (mmol/L)	103.0 (99.0, 107.0)	103.0 (99.0, 107.0)	0.018
**Vital signs**			
Temperature (°C)	37.1 (36.6, 37.6)	37.0 (36.6, 37.5)	0.023
MAP, (mmHg)	75.7 (69.1, 83.1)	75.9 (70.6, 83.0)	0.049
Heart rate, (min^, −1^)	90.6 (78.9, 102.8)	90.1 (78.0, 101.9)	0.014
Respiratory rate, (min^, −1^)	19.6 (16.2, 22.9)	19.4 (17.0, 22.7)	0.013
**Comorbidities**, ***n*** **(%)**			
Hypertension	225 (34.5%)	227 (34.8%)	0.013
DM	173 (26.5%)	187 (28.7%)	0.020
CHD	108 (16.6%)	113 (17.3%)	0.004
COPD	12 (1.8%)	12 (1.8%)	0.000
CKD	68 (10.4%)	77 (11.8%)	0.010
**Source of infection**, ***n*** **(%)**			
Lung	441 (67.6%)	436 (66.9%)	0.000
Abdomen	66 (10.1%)	67 (10.3%)	0.031
Urine	261 (40.0%)	267 (41.0%)	0.013
Soft tissue	32 (4.9%)	29 (4.4%)	0.025
Central nervous system	8 (1.2%)	6 (0.9%)	0.014
**Treatment**			
Vasopressor	234 (3.6%)	244 (3.7%)	0.032
Mechanical ventilation	506 (77.6%)	506 (77.6%)	0.000
RRT	30 (4.6%)	29 (4.4%)	0.007
**Severity scales**			
SOFA	6 (4, 9)	6 (4, 9)	0.006
SAPSII	42 (34, 54)	43 (33, 52)	0.002

*Values were shown as median (interquartile range) unless otherwise indicated*.

*WBC, white blood cell; INR, international normalized ratio; PTT, partial thromboplastin time; Scr, serum creatitine; PO_2_, partial pressure of oxygen; PCO_2_, partial pressure of carbon dioxide; MAP, mean arterial pressure; DM, diabetes mellitus; CHD, coronary heart disease; COPD, chronic obstructive pulmonary disease; CKD, chronic renal disease; RRT, renal replacement therapy; SMD, standardized mean difference; SOFA, Sequential Organ Failure Assessment; SAPSII, Simplified Acute Physiology Score II*.

### Subgroup Analysis

**Figure 3:**
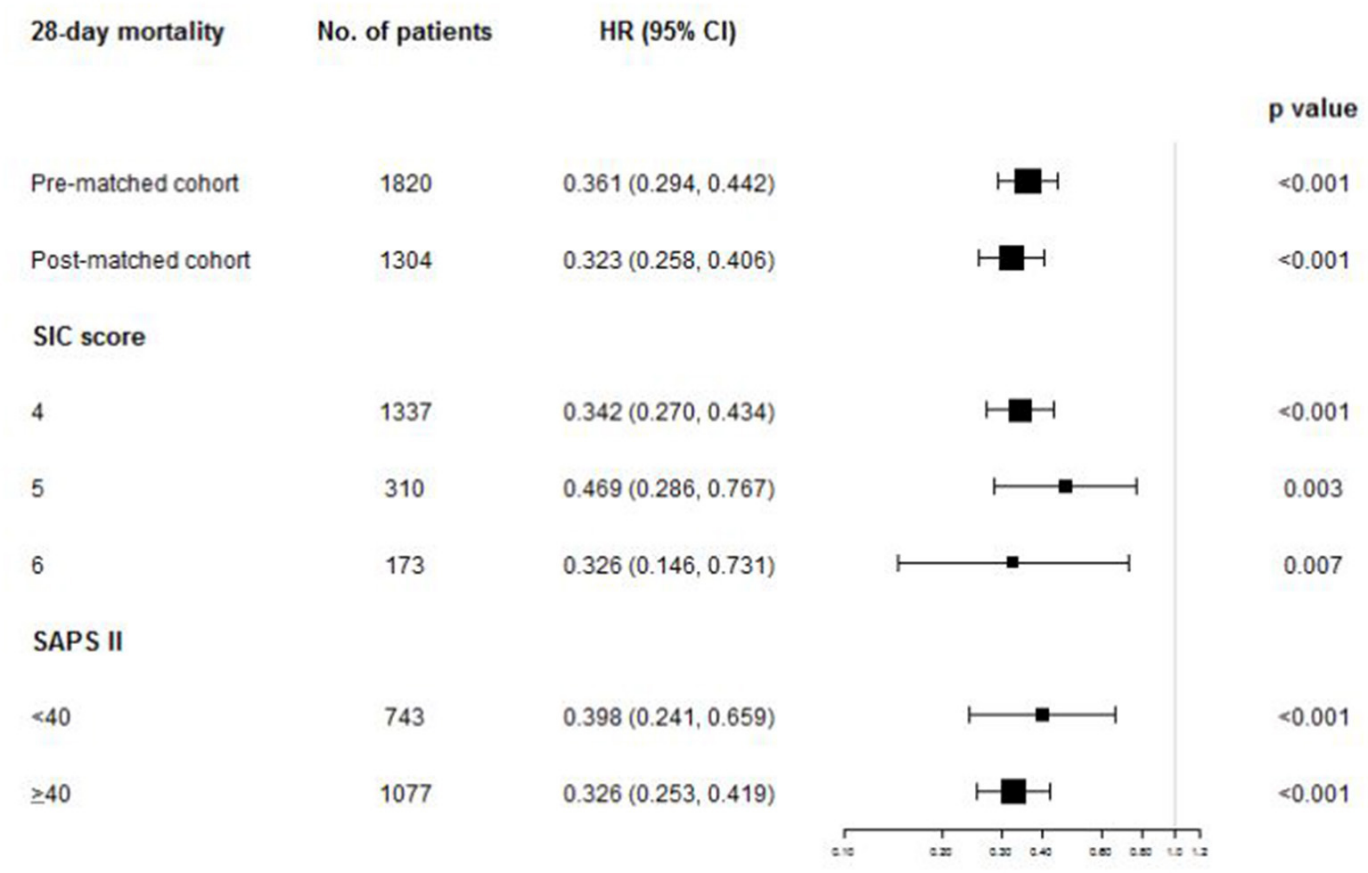
The association between UFH administration and 28-day mortality in overall population and subgroups. *HR, hazard ratio; CI, confidence interval; SIC, sepsis-induced coagulopathy; SAPS II, Simplified Acute Physiology Score II*.

No matter stratified by SIC score or SAPS II, the 28-day survival analysis showed that patients in the UFH group had ongoing lower mortality compared with the control group in all the subgroups (all *p* < 0.001) (Additional file 1: Supplementary Figures 2, 3). The multivariate Cox-proportional hazards models also found that the application of UFH was also significantly associated with reduced 28-day mortality in all the subgroups (Figure 3). In terms of adverse events, UFH use was associated with increased risk of intracranial hemorrhage in the subgroup of SIC score of 4 (adjusted OR, 1.980, 95% CI, 1.286–3.049; *p* = 0.002) and SAPS II <40 (adjusted OR, 2.617, 95% CI, 1.440–4.755; *p* = 0.002). Other secondary outcomes of each subset were demonstrated in detail in Additional file 1: Supplementary Tables 4–8.

## Discussion

Dysregulation of the coagulation is common in sepsis and is usually associated with multiple organ dysfunction syndrome and poor prognosis owing to microvascular thrombosis (16). Therefore, it was hypothesized that the inhibition of the over-activated coagulation cascade through anticoagulants could help to resolve the problem. Heparin, a glycosaminoglycan of variable polymer length, was first applied in the treatment of sepsis in 1966 (17). Since then, many clinical studies have been conducted to investigate the efficacy and safety of heparin in sepsis. However, the role of heparin therapy in septic patients has remained controversial in the medical literature. Some studies suggested that heparin may reduce 28-day mortality in septic patients (14, 18), while others reported no effect on 28-day mortality (6, 15, 19). The major drawback of the heparin trials was the lack of biomarker to identify the appropriate target population. The inadequate stratification of septic patients based on the coagulation activation status could partly explain the negative results of trials. What's more, DIC is still poorly recognized due to the heterogeneity of its diagnostic criteria and the feature of DIC is not exactly the same as the coagulation disorder in sepsis.

Sepsis-induced coagulopathy, a new category identifying an earlier phase of DIC, was proposed by DIC SCC in 2017. It was developed to categorize patients with “sepsis and coagulation disorders” (8). In this setting, the SOFA score is used for the diagnosis of organ dysfunction and SIC should be defined as “infection-induced organ dysfunction and coagulopathy.” Few studies have so far allocated anticoagulant treatments to a selected subset of patients based on SIC criteria. Through retrospective analysis of MIMIC-III database, 6,425/19,613 (32.7%) septic patients developed SIC and 1,820 patients were finally studied according to the exclusion criteria. Our study revealed that anticoagulation with UFH significantly reduced 28-day mortality and hospital mortality compared with the control group with adjusted HR 0.361 (95% CI, 0.294–0.442; *p* < 0.001) and 0.414 (95% CI, 0.314–0.502; *p* < 0.001), respectively. After propensity matching to the heterogeneity of baseline clinical features between the groups, it didn't change the main results of favorable outcomes by UFH administration. After stratifying patients according to the severity of coagulopathy, survival benefits were evident among all the subgroups. Some *post hoc* analysis of randomized clinical trials found that less seriously ill patients (e.g., SAPS II <40) did not benefit from anticoagulant therapy (20). Then, we further stratified patients according to severity of disease by SAPS II. In any risk stratum, mortality was significantly lower in the UFH compared with the control group.

The central concern, as with all anticoagulants, is the potential risk of major hemorrhage. Two meta-analyses reported that heparin didn't significantly increase the risk of major hemorrhage (21, 22). In our study, UFH group was associated with increased risk of intracranial hemorrhage with OR 1.933 (95% CI, 1.317–2.837; *p* < 0.001) in overall population. However, this adverse impact disappeared after PSM between the two groups. For subgroup analysis, intracranial hemorrhage was more common in patients with SIC score <4 (9.8%) and SAPS II <40 (11%) when they were prescribed with UFH. A multicenter study in Japan also found that occurrences of bleeding complications were lower in the sepsis subset with severe disease compared with the lower-risk subset when using anticoagulant therapy (23). So, our results suggested septic patients with severe disease may benefit more from anticoagulant therapy. Overall, the available evidence on safety outcomes of heparin is still insufficient and we still cannot ignore the downside of anticoagulant therapy. Thus, further research is needed to assess the harm that can be caused by this therapy.

We acknowledged several limitations of our study. First, it was a single-center retrospective analysis and hence suffers from potential selection and ascertainment bias. The baseline characteristics and intensity of ICU treatments other than anticoagulant therapies were different between the two groups. To cope with these imbalances, we applied a multivariable Cox proportional hazards regression model and propensity score matching to confirm the robustness of our findings. However, we are not confident that biased estimation of the effects can be completely excluded. Second, due to the retrospective property of this cohort study, the indications for treatment and methodology for the treatment intervention being examined were not standardized. Therefore, it is difficult for us to investigate the impact of the timing and dosage of UFH administration on mortality in all participants. Third, this study involves several subgroup analyses. Thus, the potential for accidental false-positive results cannot be denied. Fourth, it is hard to account for past medical history with high certainty. So, the results should be interpreted with caution. Finally, a prospective randomized controlled double-blind clinical trial is urgently needed to evaluate the potential benefit and safety of heparin given in various doses and routes in septic patients based on SIC criteria to further prove the results.

## Conclusion

Sepsis-associated DIC is characterized by suppression of fibrinolysis induced by endothelial dysfunction, which can develop into multiple organ failure and death in a short time. Using SIC criteria will facilitate early recognition of DIC and potentially hasten intervention in clinic. Our retrospective analysis using MIMIC-III database demonstrated an association between anticoagulant therapy with UFH and lower mortality in septic patients with sepsis-induced coagulopathy regardless of coagulopathy or disease severity. We believe that this initial investigation will further enhance study to evaluate the efficacy of anticoagulant therapy in sepsis-induced coagulopathy.

## Data Availability Statement

The raw data supporting the conclusions of this article will be made available by the authors, without undue reservation.

## Ethics Statement

The studies involving human participants were reviewed and approved by Institutional Review Boards of the Massachusetts Institute of Technology and Beth Israel Deaconess Medical Center. Written informed consent for participation was not required for this study in accordance with the national legislation and the institutional requirements.

## Author Contributions

J-CP wrote the manuscript. FN analyzed the data. Y-JL did the statistical work. Q-YX extracted the data. S-PX designed the study. WL participated in coordination. YG reviewed and revised the manuscript. All authors read and approved the final manuscript.

## Funding

This work was supported by the grants from Science and Technology Committee of Shanghai Municipality [Grant Number 18411951100].

## Conflict of Interest

The authors declare that the research was conducted in the absence of any commercial or financial relationships that could be construed as a potential conflict of interest.

## Publisher's Note

All claims expressed in this article are solely those of the authors and do not necessarily represent those of their affiliated organizations, or those of the publisher, the editors and the reviewers. Any product that may be evaluated in this article, or claim that may be made by its manufacturer, is not guaranteed or endorsed by the publisher.

## Abbreviations

CI: confidence interval
CKD: chronic kidney disease
CHD: chronic heart disease
COPD: chronic obstructive pulmonary disease
DIC: disseminated intravascular coagulation
DM: diabetes mellitus
eGFR: estimated glomerular filtration rate
HR: hazard ratio
ICD: International Classification of Diseases
ISTH: International Society of Thrombosis and Haemostasis
INR: international normalized ratio
IPTW: inverse probability of treatment weighing
IQR: interquartile range
JAAM: Japanese Association for Acute Medicine
LMWH: Low Molecular Weight Heparin
MAP: mean arterial pressure
MIMIC: Medical Information Mart for Intensive Care
OR: odds ratio
PSM: Propensity score matching
PT: prothrombin time
PTT: partial thromboplastin time
RRT: renal replacement therapy
SAPS II: Simplified Acute Physiology Score II
Scr: serum creatinine
SD: standard deviation
SIC: sepsis-induced coagulopathy
SMD: Standardized mean difference
SOFA: Sequential Organ Failure Assessment
SSC: Scientific and Standardization Committee
TB: total bilirubin
UFH: unfractioned heparin
WBC: white blood cell.
