# Supplementary material for: Favorable Outcomes of Anticoagulation With Unfractioned Heparin in Sepsis-Induced Coagulopathy: A Retrospective Analysis of MIMIC-III Database
Source: Front Med (Lausanne). 2022 Jan 3;8:773339. doi: 10.3389/fmed.2021.773339 (PMC8761617; doi:10.3389/fmed.2021.773339)
Supplement: Supplementary file 1 [file Data_Sheet_1.docx]

Table S1. Sepsis‐induced coagulopathy criteria

|  | Score | Range |
| --- | --- | --- |
| Platelet count (10^3^/μL) | 2 | < 100 |
|  | 1 | ≧ 100, < 150 |
| INR | 2 | > 1.4 |
|  | 1 | > 1.2, ≦1.4 |
| SOFA score | 2 | ≧ 2 |
|  | 1 | 1 |
| Total score for SIC |  | ≧ 4 |

SIC sepsis‐induced coagulopathy, INR international normalized ratio, SOFA sequential organ failure assessment.

Table S2. Missing number for risk variables and outcome variables

| **Risk Variables** | Missing number (%) |
| --- | --- |
| Age | 0 (0) |
| Gender | 0 (0) |
| WBC | 0 (0) |
| Hemoglobin | 0 (0) |
| Platelet | 0 (0) |
| INR | 0 (0) |
| PTT | 73 (4.0%) |
| Albumin | 455 (25%) |
| Bilirubin | 388 (21.3) |
| Scr | 0 (0) |
| pH | 187 (10.3%) |
| PO_2_ | 187 (10.3%) |
| PCO_2_ | 187 (10.3%) |
| Lactate | 499 (27.4) |
| Anion gap | 19 (1.0%) |
| Bicarbonate | 5 (0.3%) |
| Sodium | 0 (0) |
| Potassium | 0 (0) |
| Chloride | 0 (0) |
| Temperature | 44 (2.4%) |
| MAP | 11 (0.6%) |
| Heart rate | 11 (0.6%) |
| Respiratory rate | 11 (0.6%) |
| Hypertension | 0 (0) |
| DM | 0 (0) |
| CHD | 0 (0) |
| COPD | 0 (0) |
| CKD | 0 (0) |
| Vasopressor | 0 (0) |
| Mechanical ventilation | 0 (0) |
| RRT | 0 (0) |
| SOFA | 0 (0) |
| SAPSII | 0 (0) |
| **Outcome variables** |  |
| 28-day mortality | 0 (0) |
| Hospital mortality | 0 (0) |
| Length of ICU stay | 0 (0) |
| Length of hospital stay | 0 (0) |
| Intracranial haemorrhage | 0 (0) |
| Gastrointestinal bleeding | 0 (0) |

WBC, white blood cell; INR, international normalized ratio; PTT, partial thromboplastin time; Scr, serum creatitine; PO_2_, partial pressure of oxygen; PCO_2_, partial pressure of carbon dioxide; MAP, mean arterial pressure; DM, diabetes mellitus; CHD, coronary heart disease; COPD, chronic obstructive pulmonary disease; CKD, chronic renal disease; RRT, renal replacement therapy; SOFA, Sequential Organ Failure Assessment; SAPSII, Simplified Acute Physiology Score II.

Table S3. Univariate analysis of Cox-proportional hazards model for risk of 28-day mortality.

| Variables | HR | 95% CI | *p* value |
| --- | --- | --- | --- |
| Age | 1.030 | (1.023, 1.036) | <0.001 |
| Male | 1.070 | (0.899, 1.273) | 0.446 |
| **Laboratory tests** | | | |
| WBC | 1.006 | (1.003, 1.010) | <0.001 |
| Hemoglobin | 0.982 | (0.935, 1.032) | 0.479 |
| Platelet | 0.992 | (0.946, 1.021) | 0.099 |
| INR | 1.091 | (1.039, 1.145) | <0.001 |
| PTT | 1.003 | (1.001, 1.005) | 0.035 |
| Albumin | 0.920 | (0.782, 1.082) | 0.315 |
| Bilirubin | 1.015 | (0.997, 1.033) | 0.1082 |
| Scr | 1.087 | (1.039, 1.137) | <0.001 |
| pH | 0.271 | (0.132, 0.559) | <0.001 |
| PO_2_ | 0.929 | (0.887, 0.985) | 0.001 |
| PCO_2_ | 1.004 | (0.997, 1.010) | 0.252 |
| Lactate | 1.071 | (1.034, 1.108) | <0.001 |
| Anion gap | 1.052 | (1.036, 1.068) | <0.001 |
| Bicarbonate | 0.967 | (0.951, 0.983) | <0.001 |
| Sodium | 1.011 | (0.994, 1.027) | 0.205 |
| Potassium | 1.034 | (0.957, 1.117) | 0.399 |
| Chloride | 0.987 | (0.974, 0.999) | 0.039 |
| **Vital signs** | | | |
| Temperature | 1.312 | (1.282, 1.324) | <0.001 |
| MAP | 0.986 | (0.978, 0.994) | 0.001 |
| Heart rate | 0.993 | (0.988, 0.998) | 0.005 |
| Respiratory rate | 1.036 | (1.019, 1.053) | <0.001 |
| **Comorbidities** | | | |
| Hypertension | 1.097 | (0.914, 1.317) | 0.321 |
| DM | 0.920 | (0.753, 1.124) | 0.414 |
| CHD | 1.046 | (0.839, 1.304) | 0.690 |
| COPD | 1.533 | (0.917, 2.562) | 0.103 |
| CKD | 1.468 | (1.162, 1.855) | 0.001 |
| **Source of infection** | | | |
| Lung | 1.154 | (0.954, 1.395) | 0.139 |
| Abdomen | 0.683 | (0.572, 0.817) | <0.001 |
| Urine | 0.485 | (0.299, 0.786) | 0.003 |
| Soft tissue | 0.530 | (0.198, 1.418) | 0.206 |
| Central nervous system | 0.773 | (0.580, 1.030) | 0.079 |
| **Treatment** | | | |
| Vasopressor | 1.776 | （1.494, 2.111） | <0.001 |
| Mechanical ventilation | 0.901 | （0.742, 1.093） | 0.289 |
| RRT | 1.233 | （0.882, 1.722） | 0.220 |
| **Severity scales** | | | |
| SOFA | 1.070 | (1.044, 1.097) | <0.001 |
| SAPSII | 1.030 | (1.025, 1.035) | <0.001 |

WBC, white blood cell; INR, international normalized ratio; PTT, partial thromboplastin time; Scr, serum creatitine; PO_2_, partial pressure of oxygen; PCO_2_, partial pressure of carbon dioxide; MAP, mean arterial pressure; DM, diabetes mellitus; CHD, coronary heart disease; COPD, chronic obstructive pulmonary disease; CKD, chronic renal disease; RRT, renal replacement therapy; SOFA, Sequential Organ Failure Assessment; SAPSII, Simplified Acute Physiology Score II.

Table S4. Sensitivity analysis for patients with SIC score of 4

| **Clinical outcomes** | Control group  (n=743) | UFH group  (n=594) | *p* value | Effect size (95% CI) | *p* value |
| --- | --- | --- | --- | --- | --- |
| 28-day mortality^a^ | 259 (34.9%) | 98 (16.5%) | <0.001 | HR=0.342  (0.270, 0.434) | <0.001 |
| Hospital mortality^a^ | 259 (34.9%) | 113 (19.0%) | <0.001 | HR=0.369  (0.294, 0.463) | <0.001 |
| Length of ICU stay, days^b^ | 6.6 (4.2, 12.0) | 11.2 (6.9, 18.8) | <0.001 | β=4.700  (3.609, 5.791) | <0.001 |
| Length of hospital stay, days ^b^ | 13.4 (7.6, 22.1) | 17.2 (12.4, 26.8) | <0.001 | β=4.506  (2.945, 6.068) | <0.001 |
| Intracranial haemorrhage^c^ | 38 (5.1%) | 58 (9.8%) | 0.001 | OR=1.980  (1.286, 3.049) | 0.002 |
| Gastrointestinal bleeding^c^ | 11 (1.5%) | 16 (2.7%) | 0.117 | OR=1.823  (0.830, 4.006) | 0.135 |

Values are shown as median (interquartile range) or n (%) unless otherwise indicated.

^a^ Cox regression was used for estimating the impact of UFH use on mortality outcomes after adjusting for confounding variables selected based on *p* value < 0.05 in univariate analysis. Results were given as hazard ratio (HR) and 95% confidence interval (CI).

^b^ Linear regression model was used to evaluate the impact of UFH use on length of stay after adjusting for age, gender, SOFA score and SAPSII. Results were given as beta coefficient and 95% CI.

^c^ Bivariate logistic regression was used to assess the associations between UFH use and bleeding complications after adjusting for age, gender, platelet count, INR, PTT, SOFA score and SAPSII. Results were given as odds ratio (OR) and 95% CI.

Table S5. Sensitivity analysis for patients with SIC score of 5

| **Clinical outcomes** | Control group  (n=197) | UFH group  (n=113) | *p* value | Effect size (95% CI) | *p* value |
| --- | --- | --- | --- | --- | --- |
| 28-day mortality^a^ | 75 (38.1%) | 22 (19.5%) | 0.001 | HR=0.469  (0.286, 0.767) | 0.003 |
| Hospital mortality^a^ | 82 (41.6%) | 21 (21.2%) | <0.001 | HR=0.486  (0.304, 0.787) | 0.003 |
| Length of ICU stay, days^b^ | 6.6 (4.2, 11.8) | 10.1 (5.7, 15.7) | <0.001 | β=2.285  (0.413, 4.158) | 0.017 |
| Length of hospital stay, days ^b^ | 13.3 (8.2, 22.0) | 16.4 (10.5, 23.5) | 0.018 | β=0.457  (-3.225, 4.140) | 0.807 |
| Intracranial haemorrhage^c^ | 10 (5.1%) | 9 (8.0%) | 0.308 | OR=1.962  (0.717, 5.367) | 0.189 |
| Gastrointestinal bleeding^c^ | 4 (2.0%) | 1 (0.9%) | 0.656 | OR=1.609  (0.096, 26.939) | 0.741 |

Values are shown as median (interquartile range) or n (%) unless otherwise indicated.

^a^ Cox regression was used for estimating the impact of UFH use on mortality outcomes after adjusting for confounding variables selected based on *p* value < 0.05 in univariate analysis. Results were given as hazard ratio (HR) and 95% confidence interval (CI).

^b^ Linear regression model was used to evaluate the impact of UFH use on length of stay after adjusting for age, gender, SOFA score and SAPSII. Results were given as beta coefficient and 95% CI.

^c^ Bivariate logistic regression was used to assess the associations between UFH use and bleeding complications after adjusting for age, gender, platelet count, INR, PTT, SOFA score and SAPSII. Results were given as odds ratio (OR) and 95% CI.

Table S6. Sensitivity analysis for patients with SIC score of 6

| **Clinical outcomes** | Control group  (n=129) | UFH group  (n=44) | *p* value | Effect size (95% CI) | *p* value |
| --- | --- | --- | --- | --- | --- |
| 28-day mortality^a^ | 55 (42.6%) | 8 (18.2%) | 0.004 | HR=0.326  (0.146, 0.731) | 0.007 |
| Hospital mortality^a^ | 66 (51.2%) | 11 (25.0%) | 0.003 | HR=0.462  (0.230, 0.929) | 0.030 |
| Length of ICU stay, days^b^ | 7.3 (4.7, 13.5) | 14.8 (9.8, 23.3) | <0.001 | β=6.394  (2.258, 10.529) | 0.001 |
| Length of hospital stay, days ^b^ | 15.4 (8.6, 27.3) | 24.6 (14.3, 35.2) | 0.002 | β=3.294  (-3.918, 10.485) | 0.369 |
| Intracranial haemorrhage^c^ | 2 (1.6%) | 1 (2.3%) | 0.751 | OR=1.613  (0.090, 28.764) | 0.745 |
| Gastrointestinal bleeding^c^ | 4 (3.1%) | 0 (0.0%) | 0.573 | / | / |

Values are shown as median (interquartile range) or n (%) unless otherwise indicated.

^a^ Cox regression was used for estimating the impact of UFH use on mortality outcomes after adjusting for confounding variables selected based on *p* value < 0.05 in univariate analysis. Results were given as hazard ratio (HR) and 95% confidence interval (CI).

^b^ Linear regression model was used to evaluate the impact of UFH use on length of stay after adjusting for age, gender, SOFA score and SAPSII. Results were given as beta coefficient and 95% CI.

^c^ Bivariate logistic regression was used to assess the associations between UFH use and bleeding complications after adjusting for age, gender, platelet count, INR, PTT, SOFA score and SAPSII. Results were given as odds ratio (OR) and 95% CI.

Table S7. Sensitivity analysis for patients with SASP II < 40

| **Clinical outcomes** | Control group  (n=434) | UFH group  (n=309) | *p* value | Effect size (95% CI) | *p* value |
| --- | --- | --- | --- | --- | --- |
| 28-day mortality^a^ | 85 (19.6%) | 27 (8.7%) | <0.001 | HR=0.398  (0.241, 0.659) | <0.001 |
| Hospital mortality^a^ | 86 (19.8%) | 27 (8.7%) | <0.001 | HR=0.380  (0.231, 0.624) | <0.001 |
| Length of ICU stay, days^b^ | 6.5 (4.4, 11.3) | 10.2 (5.5, 17.1) | <0.001 | β=3.609  (2.168, 5.050) | <0.001 |
| Length of hospital stay, days ^b^ | 14.6 (8.7, 24.2) | 17.4 (12.5, 26.6) | <0.001 | β=3.089  (1.910, 5.268) | 0.006 |
| Intracranial haemorrhage^c^ | 19 (4.4%) | 3.4 (11.0%) | 0.001 | OR=2.617  (1.440, 4.755) | 0.002 |
| Gastrointestinal bleeding^c^ | 5 (1.2 %) | 2 (0.6%) | 0.706 | OR=0.414  (0.076, 2.258) | 0.308 |

Values are shown as median (interquartile range) or n (%) unless otherwise indicated.

^a^ Cox regression was used for estimating the impact of UFH use on mortality outcomes after adjusting for confounding variables selected based on *p* value < 0.05 in univariate analysis. Results were given as hazard ratio (HR) and 95% confidence interval (CI).

^b^ Linear regression model was used to evaluate the impact of UFH use on length of stay after adjusting for age, gender, SOFA score and SAPSII. Results were given as beta coefficient and 95% CI.

^c^ Bivariate logistic regression was used to assess the associations between UFH use and bleeding complications after adjusting for age, gender, platelet count, INR, PTT, SOFA score and SAPSII. Results were given as odds ratio (OR) and 95% CI.

Table S8. Sensitivity analysis for patients with SASP II ≥ 40

| **Clinical outcomes** | Control group  (n=635) | UFH group  (n=442) | *p* value | Effect size (95% CI) | *p* value |
| --- | --- | --- | --- | --- | --- |
| 28-day mortality^a^ | 304 (47.9%) | 101 (22.9%) | <0.001 | HR=0.326  (0.253, 0.419) | <0.001 |
| Hospital mortality^a^ | 321 (50.6%) | 121 (27.4%) | <0.001 | HR=0.368  (0.291, 0.466) | <0.001 |
| Length of ICU stay, days^b^ | 7.8 (4.6, 12.5) | 11.4 (7.3, 19.9) | <0.001 | β=5.064  (3.832, 6.297) | <0.001 |
| Length of hospital stay, days ^b^ | 13.8 (7.7, 21.9) | 17.8 (11.3, 27.6) | <0.001 | β=3.981  (2.067, 5.895) | <0.001 |
| Intracranial haemorrhage^c^ | 31 (4.9%) | 34 (7.7%) | 0.001 | OR=1.580  (0.950, 2.627) | 0.078 |
| Gastrointestinal bleeding^c^ | 14 (2.2 %) | 15 (3.4%) | 0.236 | OR=1.251  (0.599, 2.612) | 0.552 |

Values are shown as median (interquartile range) or n (%) unless otherwise indicated.

^a^ Cox regression was used for estimating the impact of UFH use on mortality outcomes after adjusting for confounding variables selected based on *p* value < 0.05 in univariate analysis. Results were given as hazard ratio (HR) and 95% confidence interval (CI).

^b^ Linear regression model was used to evaluate the impact of UFH use on length of stay after adjusting for age, gender, SOFA score and SAPSII. Results were given as beta coefficient and 95% CI.

^c^ Bivariate logistic regression was used to assess the associations between UFH use and bleeding complications after adjusting for age, gender, platelet count, INR, PTT, SOFA score and SAPSII. Results were given as odds ratio (OR) and 95% CI.


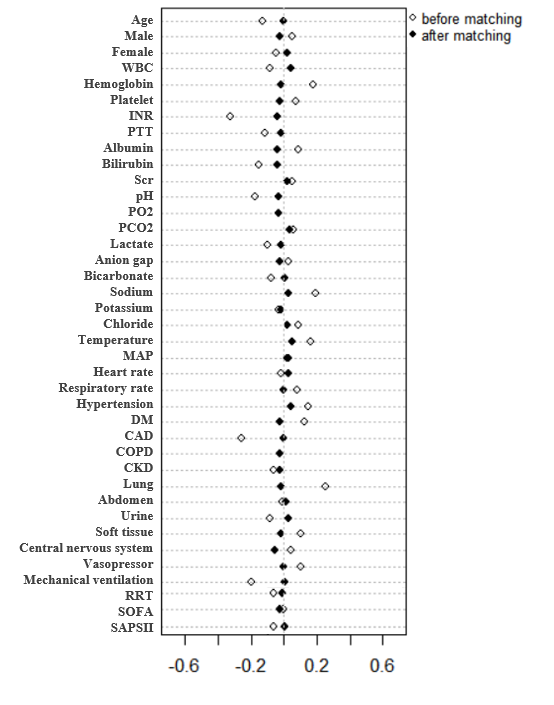


Figure S1. Standardized mean difference (SMD) of variables before and after propensity score matching.


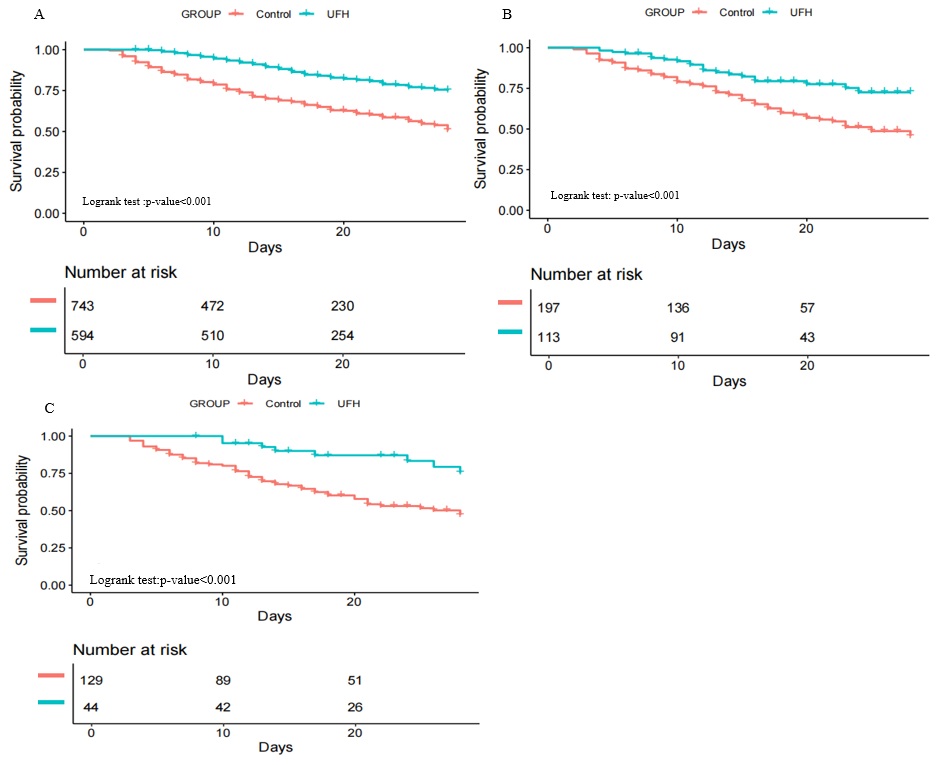


Figure S2. Kaplan–Meier estimates of cumulative probabilities of 28-day survival for patients with SIC score of 4 (A), 5 (B) and 6 (C) in the UFH group and the control group.


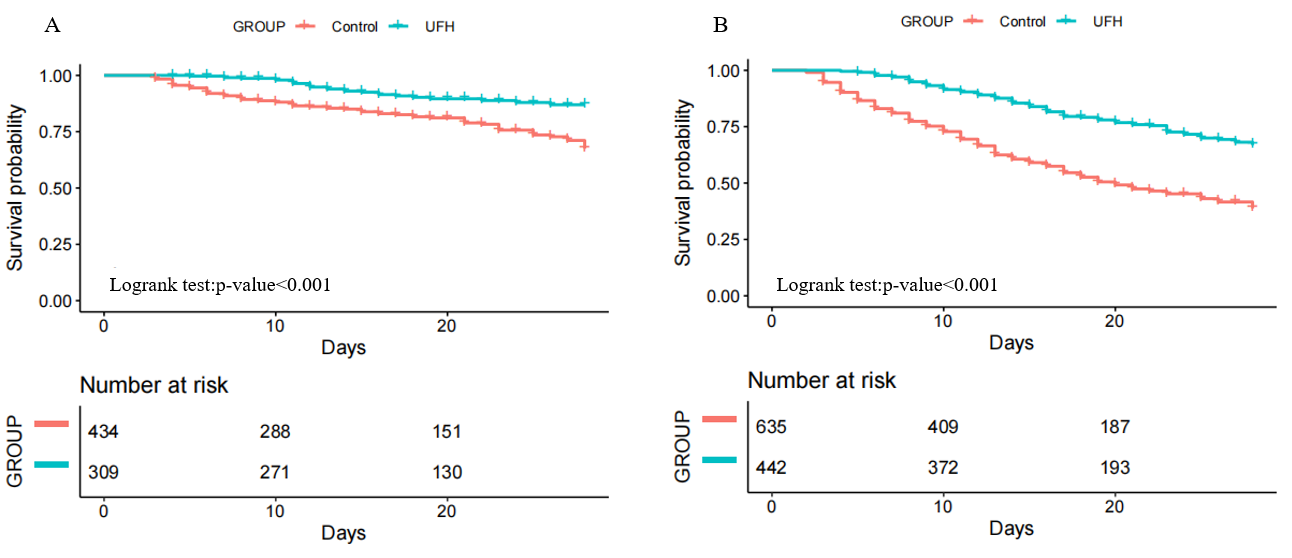


Figure S3. Kaplan–Meier estimates of cumulative probabilities of 28-day survival for patients with SAPS II < 40 (A) and SAPS II ≥ 40 (B) between the UFH group and the control group.
